# Adjuvant-dependent cytokine profiles in the context of a DNA prime-protein boost HIV-1 vaccine

**DOI:** 10.1186/1742-4690-9-S2-P16

**Published:** 2012-09-13

**Authors:** R Buglione-Corbett, K Pouliot, R Marty-Roix, K West, S Wang, E Lien, S Lu

**Affiliations:** 1 University of Massachusetts Medical School, Worcester, MA, USA

## Background

Heterologous prime-boost vaccinations have emerged as a promising strategy to generate protective immunity against a variety of pathogens. Our previous clinical work has demonstrated that an HIV-1 gp120 DNA prime-protein boost vaccine, DP6-001, elicits enhanced neutralizing antibody responses as well as cell-mediated immune responses in humans. However, the roles of adjuvants remain largely unknown in the context of such combination vaccines.

## Methods

In a mouse model, we studied the effects of adjuvants QS-21, Alum, and MPL, in the context of DP6-001. Both gp120-specific antibody and T cell responses were monitored by ELISA and intracellular cytokine staining (ICS), respectively. Innate cytokine profiles were determined in sera collected 6 hours post-immunization by Cytometric Bead Array (CBA) and Luminex assays.

## Results

Serum anti-Env IgG titers were comparable between adjuvant groups. All immunized animals demonstrated comparable positive gp120-specific CD4+ and CD8+ T cell responses by ICS. Adjuvant profiles were largely determined by sera cytokines following protein boosting. QS-21 was distinguished by elevated IL-4, IFNγ, MIP-1β, and IL-1β. MPL was characterized by elevated G-CSF, KC, and RANTES. Both adjuvant groups demonstrated elevated IL-6. Production of these sera cytokine profiles required DNA priming.

## Conclusion

Our data indicated that different adjuvants generate unique patterns of biomarkers, indicating that different mechanisms are involved in their action. Our results also provided useful guidance in the selection of an adjuvant for inclusion in future prime-boost strategies, with the goal of enhancing immunogenicity while minimizing reactogenicity.

